# Characteristics of Cognitive in Children with Learning Difficulties

**DOI:** 10.1515/tnsci-2019-0024

**Published:** 2019-05-21

**Authors:** Feng Liang, Panpan Li

**Affiliations:** 1 College of Healthy Management, Shangluo University, Shangluo, Shaanxi 726000, China

**Keywords:** Characteristics of Cognitive, Children, Learning Disabilities

## Abstract

In order to explore the relationship between cognitive function in children with learning difficulties and social environment, this study uses the Wechsler Intelligence Scale and the self-made general environment questionnaire to investigate 185 children with learning difficulties and compares them with 185 normal children, and gives attention test to 50 children with learning difficulties. The results show that family environment has a certain influence on the children with learning difficulties, they have a significantly lower verbal intelligence quotient (VIQ), performance intelligence quotient (PIQ) and full scale intelligence quotient (FIQ), and the separation of VIQ and P IQ is common among them. As the children with learning difficulties grow older, their ability for abstract generalization tends to decline, which may be a characteristic of their intelligence development. This study aims to compare the functional differences in cortical regions between children with learning difficulties and children without from the perspective of cognitive neuropsychology, so as to provide effective assistance for children with learning difficulties.

## 1 Introduction

**Figure 1:**
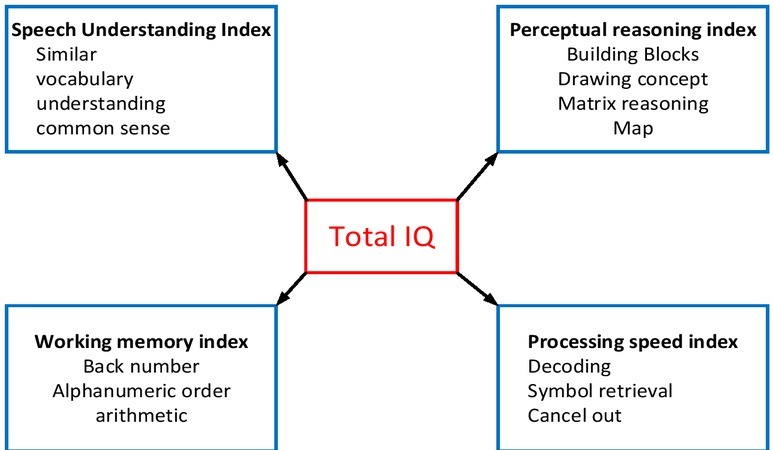
The structure of children’s cognitive function tests

Due to the development of society and the improvement of system, the problem of children with learning difficulties has become the concern to society, teachers and parents [1, 2, 3]. The research fields of many subjects deal with the problem of learning difficulty. Domestic researches mainly focus on the cognitive development, social adaptation and neurophysiology of children with learning difficulties, use some questionnaires and scales to evaluate the problems of children with learning difficulties in the above aspects, and finally put forward some strategies to solve these problems. These researches cover the definition and criteria of learning difficulty, its causes and the corresponding intervention strategies [4, 5, 6]. The high heterogeneity among children with learning difficulties has made the study of children with learning difficulties a long-term and arduous task. This study tests children with learning difficulties in a primary school in Shanghai by using a cognitive function assessment system and a screening scale for children with learning difficulties. Through statistical analysis, this study tries to reveal the characteristics of children with learning difficulties in each cognitive process, and understand the weakest parts in these cognitive processes, thereby providing help for school education. The structure of the test for children’s cognitive function is shown in Figure 1.

At present, there are many methods that can be applied to the detection of cognitive function in patients with schizophrenia. Most of the previous studies chose a single cognitive assessment scale or a combination of several simple cognitive assessment scales. Due to the lack of standardized cognitive assessment tools, the results of many different experiments cannot be well compared and summarized. This study the use of child and adolescent patients with schizophrenia to cognitive function test, can be more comprehensive understanding of the cognitive situation of patients, is relatively new cognitive tool, now has been a multinational reference and applied to clinical, Norway as the first to use the country, has proved the practicability of the test, and establish the norm of 12 ~ 59 years old.

Learning difficulties of children refer to the impairment in the acquisition or development of learning skills in school-age children who have appropriate learning opportunities, due to environmental, psychological and quality reasons, which are manifested by frequent poor academic achievements or the resulting grade retention [7]. Children with learning difficulties generally have no mental retardation. The study on children with learning difficulties began in the late 1980s, which mainly involved intelligence structure, neuropsychological characteristics, electroencephalogram (EEG) activity and family factors [8]. With a view to exploring the relationship between cognitive function of children with learning difficulties and social environment, the author conducts a comparative study on 185 children with learning difficulties and 185 normal children.

## 2 Basic situation of children with learning difficulties

### 2.1 Flaws in various cognitive features

On cognitive function in patients with schizophrenia, some scholars think that the damage might occur early in the disease, even before they can have cognitive impairment have clinical symptom in recent years at home and abroad to carry out a lot of research on cognitive function, but most of the research results are based on adult patients, for minor study of patients with few in schizophrenia patients exist obvious cognitive damage this conclusion has been recognized by most scholars and experts and confirmed that the experiment results show that, in addition to test scores no difference between the two groups of emotion management, the rest of the various test scores and total score in the two groups have significant differences. There was significant impairment in the cognitive processing speed, attention (vigilance) “working memory, speech learning and memory, visual learning and memory, reasoning and problem solving in the children and adolescents with schizophrenia in Ming dynasty.

The reason for children’s poor academic performance is that they have significant difficulties in listening, speaking, reading, writing, calculating, thinking and other

aspects of learning ability, social interaction, and behavioral adjustment [9]. But these children have a normal intelligence. This study uses a cognitive function assessment system to test children with learning difficulties, and adopts the screening scale for children with learning difficulties. For example, the electroencephalogram information of children is observed as shown in Table 1.

**Table 1:** EEG wave characteristics

	Fequency	Magnitude	Satus
α false wave	8-13Hz	20-100μV	Clear, quiet, closed eyes
β false wave	14-30Hz	50-20μV	Excited
θ false wave	4-7Hz	10-50μV	Drowsiness, sleep, lack of oxygen, depth
δ false wave	1-3.5Hz	20-200μV	Anesthetic or cerebral organic disease

Children with learning difficulties show obvious difficulties in meta-memory, working memory and short-term memory. Some studies have shown that children with learning difficulties have various degrees of difficulties in the analysis of task requirements, the selection of appropriate strategies, and allocation of learning time, monitoring and regulation of learning process, assessment results and so on.

As for the relationship between cognitive function of first-episode schizophrenia patients and negative and positive symptoms of the disease, different studies have reached different conclusions. Some researchers suggested that it was more related to negative symptoms, while others believed that it was related to positive symptoms. In this study, the EEG total score of the first episode was correlated with the negative symptom factor score, indicating that the cognitive impairment of the first episode of children and adolescents with schizophrenia was correlated with negative symptoms. This study also shows that some dimensions of cognitive function may be associated with negative or positive symptoms, but the degree of correlation is not strong, which needs to be further verified in future studies. In addition, studies have shown that the cognitive impairment of children and adolescents with schizophrenia is significantly related to gender, age and education level.

### 2.2 Behind academic achievement

Children with learning difficulties can’t remember the spelling of Chinese characters and English words, mistaken similar-looking words, jump lines or words when reading articles, and spend more time in finishing tasks such as exam [10]. Formula (1) is used to calculate the emotional value of the child w.

(1) $$o\left(w\right)\frac{1}{n}\sum_{i}sim\left(w,\;p_{i}\right)-\frac{1}{m}\sum_{j}sim\left(w,\;n_{j}\right)$$

If one or two adverbs appear in front of the emotional value of the child w, then the tendency and intensity expressed by the emotional word is calculated through Formula (2).

(2) $$o\left(w\right)=d_{1}∙d_{2}∙\left(\frac{1}{n}\sum_{i}sim\left(w,\;p_{i}\right)-\frac{1}{m}\sum_{j}sim\left(w,\;n_{j}\right)\right)$$

In comparison with normal children, children with learn difficulties have significantly low academic achievement in certain subjects, which does not match their ability. Learning difficulties in writing, reading, and mathematics may occur simultaneously in children with learning difficulties. The abilities of language learning have a greater impact on mathematics learning. In order to analyze the differences between the emotions of two children, tf-idf is used to calculate the weight of the feature term, with the calculation formula as follows.

(3) $$tf-idf\left(t_{k},\;d_{j}\right)=tf\left(t_{k},d_{j}\right)\times \log\frac{N}{n\left(t_{k}\right)}$$

Due to the differences between children’s emotions, it is generally necessary to normalize the tf-idf value to obtain the weight wkj of the feature tk in each child dj, and the calculation formula is as follows.

(4) $$w_{kj}=\frac{tf-idf\left(t_{k},\;d_{j}\right)}{\sqrt{\sum_{s}{\left(tf-idf\left(t_{s},\;d_{j}\right)\right)}^{2}}}$$

In this study, the emotional word dictionary is used to select the text feature items. The weight of the feature items is calculated by using tf - idf and emotional value of emotional words. This study improves the formula of tf - idf, as shown below.

(5) $$w_{kj}=\frac{\left(1-\lambda \right)\times tf-idf\left(t_{k},\;d_{j}\right)+\lambda o\left(t_{k}\right)}{\sqrt{\sum_{s}{\left(t\left(1-\lambda \right)\times tf-idf\left(t_{s},\;d_{j}\right)+\lambda o\left(t_{s}\right)\right)}^{2}}}$$

### 2.3 Various degrees of obstacles in social development

**Figure 2:**
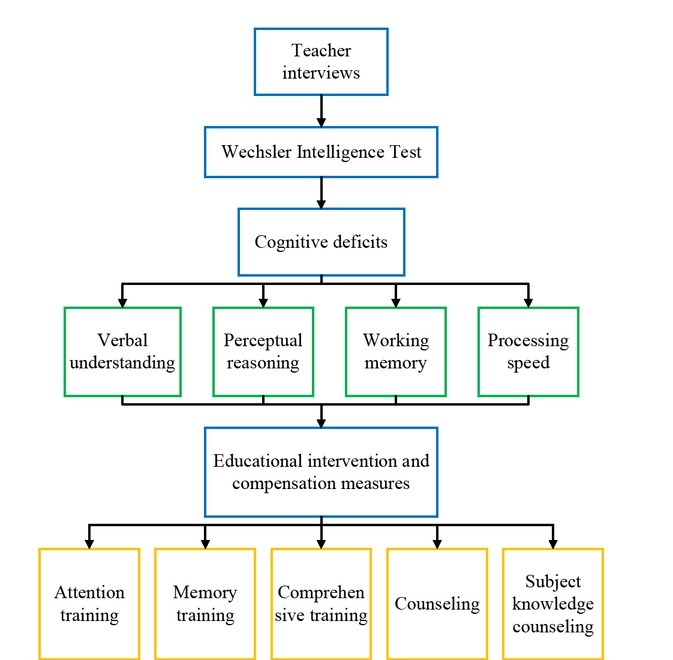
Difficulty children cognitive intervention flow chart

The poor social development of children with learning difficulties refers to inferiority and negativity in cognitive development level, low learning levels, and low self-esteem, depression, anxiety, and aggressive behavior and withdrawal behavior in moods. These children show poor performance in terms of individual independence. They usually have the following characteristics in their human relations. They usually have few friends in life, cannot fit into the groups, or do not take the initiative to speak with friends or invite friends. They usually follow others in doing things without their own opinions and are indifferent to groups. The flowchart of cognitive intervention for children with learning difficulties is shown in Figure 2.

The subjects of this study were 30 patients with schizophrenia with the first onset, who were clearly diagnosed by 2 attending psychiatrists or more and met the diagnostic criteria of schizophrenia in the international classification of diseases (icd-10). 6 years of education, able to read and understand Chinese; 11~18 years old; No antipsychotics (or less than 3d) or drugs with known cognitive effects were used prior to enrolment; 1. First onset, no more than 1 year of medical history; 2 PSS total score >60; Patients and their families had a full understanding of this study, voluntarily participated in this study, and signed the informed consent. The health group was a random selection of healthy children and adolescents aged between 11 and 18, with a > years of education, and I and my family voluntarily participated in this study, and

signed the written informed consent. Exclusion criteria: all subjects were excluded from the study for color blindness, color weakness or other cognitive disorders associated with brain organic diseases, epilepsy, mental retardation, etc. In the first group, there were 17 males and 13 females, aged from 11 to 18 years old. The control group included 17 males and 13 females, aged between 11 and 18 years. There was no significant difference between the two groups in age, sex, years of education and other demographic aspects.

## 3 Experimental Study on Cognitive Function of Children with Learning Disabilities

### 3.1 Experimental objects and methods

1. Subjects
  The group of children with learning difficulties is from the urban Shanghai, who was treated in the mental clinic of Shanghai Mental Health Center from November 1996 to November 1997. A total of 185 patients are sampled according to the order of outpatient visits, including 142 males and 43 females, with an average age of 10.2±2.1 years, and without any obvious physical and mental disorders. The criteria for defining learning difficulties are as follows: 1. The average scores of the major courses (Chinese, mathematics) are within the 10th percentile of the class. 2. The student rated as academically poor student by his head teacher according to the comprehensive assessment of his learning ability. 3. At least one major course has scored less than 60 points. 4. Intelligence quotient (IQ) > 70 points. A total of 185 children without learning difficulties are randomly selected as the control group according to the student number from a primary school in Shanghai. There are 142 males and 43 females with an average age of 10.2±2.2 years old. The selection criteria are as follows: 1. The average score of the major course is above 80 points.2. The student is rated as academically good student by his head teacher according to the comprehensive assessment of his learning ability. 3. Without any obvious physical and mental illness.
2. Research methods
  The self-made general information questionnaire is used for investigating the family environment and general psychological status of children, which is composed of 22 items in total, including maternal pregnancy, childhood development, adoption, parental education, parental relationship, parenting, lifestyle and mental status within one month before the survey, etc. The questionnaire shall be completed by the parent according to the actual situation.

### 3.2 Analysis of experimental results

The comparison of the questionnaires between the two groups show that the group with learning difficulties has many problems, such as parents’ poor educational background, improper parenting style, father’s introverted personality, emotional disorders and other behavioral problems. The results are shown in Table 2.

**Table 2:** Comparison of general environmental conditions of children with learning difficulties and normal children

	Children with learning difficulties	Normal children	X ^2^	P
I am educated at school (Dad)	133	80	31.08	<0.01
Educated in high school (Mom)	128	87	29.3	<0.01
Non-intellectuals (daddy)	126	65	40.3	<0.01
Non-intellectual (mother)	137	83	32.6	<0.01
Obedience	97	167	64.7	<0.01
Caesarean section	58	13	35.2	<0.01
Obstetric complications	30	5	19.2	<0.01
Parental education is mainly based on punishment	121	56	45.7	<0.01
Introverted father	97	61	15.4	<0.01
No interest in learning	43	15	16.3	<0.01
Mood disorders	12	2	7.4	<0.01
Will go after 2 years old	21	10	4.2	<0.01
Behavior problems	103	37	50.2	<0.01

The comparison of intelligence between the two groups shows that VIQ, PIQ and FIQ of children with learning disabilities are significantly lower than those of normal children (p < 0.01). Their score of the subscales are also lower than those of normal children, except the scores for building blocks (BD).The results are shown in Table 3. Seen from the perspective of intellectual structure, there are 95 children (51.4%) with learning difficulties that show VIQ and PIQ separation, which is significantly higher than the number in the normal group, wherein there are only 13 similar cases (7.0%) (X2=87.92p<0.01).

**Table 3:** Comparison of intelligence levels between children with learning difficulties and normal children

	Children with Learning difficulties(n=185)	Normal Children(n=185)	*t*	P
Common sense	8. 0± 2. 4	12. 6± 2. 8	- 25. 82	<0.01
Understanding	9. 4± 2. 5	10. 8± 2. 5	- 7. 99	<0.01
Arithmetic	9. 7± 2. 8	11. 4± 1. 8	- 8. 19	<0.01
Similarity	9. 3± 2. 6	12. 1± 2.	- 1 4. 73	<0.01
Vocabulary	9. 5± 2. 4	12. 2± 1. 8	- 1 5. 24	<0.01
Back number	9. 3± 2. 6	9. 5± 2. 3	- 0. 85	<0.01
Map	9. 0± 2. 7	11. 7± 2. 0	- 1 3. 45	<0.01
Building Blocks	9. 9± 2. 8	11. 3± 1. 9	- 7. 14	<0.01
Picture arrangement	8. 9± 2. 6	10. 8± 2. 3	- 1 0. 17	<0.01
Jigsaw	1 0. 9± 2. 9	10. 5± 1. 8	2. 03	<0.01
Language IQ	94. 5± 12. 2	1 10. 6± 8. 2	- 1 8. 00	<0.01
Operational IQ	97. 1± 12. 9	1 07. 0± 6. 3	- 1 0. 44	<0.01
Full scale IQ	95. 9± 11. 5	1 09. 0± 7. 0	- 1 5.	<0.01

The results of attention test of children with learning difficulties are as follows. A total of 50 children with learning difficulties have completed the attention test, including 30 males and 20 females, with an average score of 26.2±14.9, wherein there are 5 (10%) with attention ≤ 10, indicating that they do not have attention deficit, 15 (30%) with attention

ranging from 10 to 20, indicating that they may have attention deficit, and 30 (60%) with attention ≥ 20, indicating that they have attention deficit.

Among the 185 children with learning difficulties, 68 (36.8%) are diagnosed with hyperactivity disorder, and their age (9.8±2.1 years) is smaller than that of other children with learning difficulties (10.4±2.1 years) (t=-1.9, p<0.05). Among the children with learning difficulties, 164 (88.6%) can walk before 24 months old, and 21 (11.4%) after 24 months old. These children with movement retardation not only have significantly lower VIQ, PIQ, and FIQ scores than those of other children, but also the have lower scores in most subscales, as shown in Table 4.

**Table 4:** The influence of movement development on the intelligence of children with learning difficulties

	2 years old will walk(n=164)	Will go after 2 years old (n=164)	*t*	P
Understanding	9. 5± 2. 4	8. 0± 2. 3	- 2. 76	<0.01
Arithmetic	9. 9± 2. 8	8. 3± 3. 0	- 2. 46	<0.01
Similarity	9. 5± 2. 5	7. 5± 2. 9	- 3. 29	<0.01
Vocabulary	9. 7± 2. 4	8. 1± 2. 7	- 2. 48	<0.01
Back number	9. 5± 2. 5	8. 1± 2. 7	- 2. 49	<0.01
Building Blocks	10. 0± 2. 8	8. 7± 2. 4	- 2. 08	<0.01
Picture arrangement	9. 0± 2. 5	7. 8± 2. 9	- 2. 13	<0.01
Language IQ	95. 5± 1 2. 1	87. 1± 9. 9	- 3. 04	<0.01
Operational IQ	98. 2± 1 2. 6	88. 6± 1 1. 9	- 3. 30	<0.01
Full scale IQ	97. 0± 1 2. 6	87. 2± 9. 4	- 3. 82	<0.01

The research results are as follows. There are 97 cases (52.4%) of natural delivery, 58 cases (31.4%) of caesarean section, 30 cases (16.2%) with obstetric complications (such as aspiration, asphyxia, etc.). The natural delivery group score significantly higher in math, building blocks and similarities than the caesarean section group, and also score higher in similarities than the obstetric complication group. The details are shown in Table 5.

**Table 5:** The influence of different modes of delivery on the intelligence of children with learning difficulties

	Obedience (n=97)	Caesarean section (n=58)	Obstetric complications (n=30)
A	1 0. 2± 3. 0	9. 2± 2. 5	9. 9± 3. 0
S	9. 7± 2. 4	8. 7± 3. 0	8. 6± 2. 2
BD	9. 9± 2. 8	9. 0± 2. 6	10. 3± 1. 8

## Conclusions

Children’s learning difficulties are a matter of great concern to their family and society. Generally, it is generally believed that there are more than one single factors influencing children’s academic achievement, in which

intelligence level is one of the important factors and is often positively related to academic achievement. In addition, there are also family environment, learning interest, emotional state and other factors. The results of this study show that children with learning difficulties has a lower intelligence level than normal children, wherein the cases with movement retardation have a significantly lower intelligence level, and there exists attention deficit cases among children with learning difficulties, suggesting that children with learning difficulties have a biological basis. It indicates that the imbalance of intellectual structure is also an important reason for children’s learning difficulties. It shows that children with learning difficulties have an unbalanced development of speech ability and performance ability, which is shown in that their speech information processing ability obviously lags behind their non-speech processing ability.
